# Comparative roles of Wnt/PCP pathway in midline morphogenesis and cellular flows during non-amniote and amniote gastrulation

**DOI:** 10.3389/fcell.2026.1791222

**Published:** 2026-02-25

**Authors:** Rieko Asai

**Affiliations:** International Research Center for Medical Sciences, Kumamoto University, Kumamoto, Japan

**Keywords:** cellular flow, convergent extension, gastrulation, midline patterning, Wnt/PCP

## Abstract

The Wnt/Planar cell polarity (PCP) pathway is evolutionarily conserved and plays crucial roles in coordinating collective cell behaviors during embryonic development. Across the animal kingdom, the bilateral body plan is built upon the midline, whose formation begins during gastrulation, a stage characterized by large-scale cellular flows (extensive collective cell movements). In non-amniotes, midline morphogenesis is tightly coupled to these cellular flows, and this coupling is mediated by the Wnt/PCP pathway. By contrast, during amniote embryogenesis, the Wnt/PCP pathway is essential for morphogenesis of the initial midline structure, the primitive streak, particularly in avian embryos. However, its role in regulating cellular flows during primitive streak development has yet to be fully elucidated. This review integrates historical and recent findings on the Wnt/PCP pathway in midline morphogenesis and cellular flows across non-amniotes and amniotes, with a particular focus on amniote (especially avian) primitive streak development. Conserved mechanisms and species-specific developmental processes are highlighted, and the interface between the Wnt/PCP pathway and collective cell behaviors is discussed in the context of vertebrate body-plan patterning.

## 1 Introduction

Vertebrates exhibit a bilaterally symmetric body plan while developing left–right (LR) asymmetric internal organs (Martindale et al., 2002; Namigai et al., 2014). Such bilaterality is established and maintained by the midline, which defines the LR axis and coordinates patterning with the anterior–posterior (AP) and dorsal–ventral (DV) axes (Meinhardt, 2006; Bénazéraf and Pourquié, 2013). Subsequent LR laterality along the midline is specified by asymmetric expression of LR regulatory genes, including sonic hedgehog (Shh) and nodal (Levin et al., 1995; Tabin, 2006). Disruption of midline formation or errors in LR patterning can result in congenital disorders such as heterotaxy (Aylsworth, 2001; Ramsdell et al., 2006; Deng et al., 2015). Although the midline is widely conserved among bilaterians and is indispensable for establishing the bilateral body plan, the mechanisms that build and stabilize this axis remain incompletely understood.

A major molecular mechanism coordinating midline morphogenesis across vertebrates is the Wnt/Planar cell polarity (PCP) pathway, an evolutionarily conserved non-canonical Wnt signaling pathway that coordinates cytoskeletal remodeling and oriented cell behaviors, thereby establishing planar polarization within tissues (Yang and Mlodzik, 2015). In gastrulation, Wnt/PCP links molecular polarity to collective cell behaviors that contribute to shaping the emerging midline (Tada et al., 2002; Voiculescu et al., 2007; Roszko et al., 2009).

One prominent Wnt/PCP-dependent collective behavior is convergent extension (CE), the coordinated narrowing and elongation of tissues driven largely by mediolateral intercalation and other polarized rearrangements (Keller et al., 2000). In vertebrates, core PCP components become asymmetrically organized within cells, and disruption of PCP signaling, including by dominant-negative *Dishevelled* (*Dvl*) constructs that selectively impair PCP, compromises CE in frog and avian systems (Sokol, 1996; Wallingford et al., 2000; Voiculescu et al., 2007). Mechanistically, PCP engages Rho–ROCK and related small-GTPase pathways to organize actin dynamics and contractility; consistent with this, PCP inhibition can block stable bipolar protrusions in *Xenopus* notochord cells and arrest tissue elongation (Wallingford et al., 2002; Shindo, 2018). Overall, the Wnt/PCP pathway regulates CE by polarizing protrusive and junctional behaviors and coupling these cell-scale processes to tissue-scale morphogenetic processes, thereby promoting midline formation.

Midline formation begins during gastrulation, a stage characterized by large-scale cellular flows (extensive collective cell movements). In non-amniotes (e.g., fish and amphibians), midline morphogenesis is tightly coupled to these flows, and Wnt/PCP-mediated planar polarization provides a mechanistic basis for this coupling through CE and tissue elongation (Keller et al., 2000; Tada et al., 2002; Roszko et al., 2009). This tight coupling offers a relatively direct framework for connecting pathway activity to tissue-level morphogenesis.

By contrast, amniotes establish the earliest midline structure through the primitive streak (PS) in an epithelial epiblast, most prominently characterized in avian embryos (Mikawa et al., 2004). The Wnt/PCP pathway is essential for PS morphogenesis and extension (Voiculescu et al., 2007; Asai et al., 2024), yet how it contributes to the accompanying cellular flows during PS development has yet to be fully elucidated. Existing perturbation and quantitative imaging studies indicate that key aspects of tissue-scale flows can persist even when PS morphology is compromised (Asai et al., 2024), underscoring the need for systematic, quantitative tests of how PCP activity relates to flow parameters and to early LR bias prior to a mature organizer.

Accordingly, this Mini Review integrates historical and recent findings on Wnt/PCP in non-amniote and amniote gastrulation, highlights conserved mechanisms and species-specific developmental processes, and outlines testable hypotheses for how Wnt/PCP interfaces with cellular flows to establish the vertebrate body plan. We emphasize where evidence supports tight coupling, where it instead points to partial decoupling, and which quantitative measurements are needed to resolve the remaining open questions.

## 2 Conserved components of Wnt/PCP in planar polarization and collective behaviors

The Wnt/PCP pathway has been extensively studied as a conserved planar polarity machinery that is redeployed in distinct tissue contexts to bias cell behaviors and thereby organize morphogenesis. Core PCP components include Frizzled (Fz), Van Gogh-like (Vangl), Flamingo/Celsr, Prickle (Pk), and Dvl (Wansleeben and Meijlink, 2011; Gao, 2012; Davey and Moens, 2017). Through intercellular feedback and asymmetric subcellular distributions, PCP establishes planar polarity fields that can be read out by cytoskeletal regulators and junctional remodeling (Seifert and Mlodzik, 2007; Koca et al., 2022). In vertebrate development, PCP-dependent outputs include mediolateral intercalation, oriented cell elongation, and epithelial rearrangements that underlie convergent extension (Roszko et al., 2009).

Importantly, PCP acts within a broader tissue context. In some model animals, large-scale cellular flows appear to emerge as integrated outcomes of PCP-biased cell rearrangements (Tada and Smith, 2000; Wallingford et al., 2000; Matsui et al., 2005). In others, coherent flows can arise at the epithelium scale and are then shaped, or only partially constrained, by PCP-dependent remodeling (Asai et al., 2024; Zhao et al., 2025). Distinguishing between these regimes is essential for a comparative understanding of gastrulation in non-amniotes and amniotes. In this view, PCP translates molecular asymmetries into polarized cell behaviors that can scale up to coordinated tissue-level movements.

## 3 Non-amniotes: Wnt/PCP-coordinated collective behaviors couple flows and midline elongation

### 3.1 Midline morphogenesis and proliferative requirements

In non-amniotes, the embryonic axial tissues, including the forming notochord, elongate during gastrulation largely through convergent extension, driven by coordinated neighbor exchanges that narrow and lengthen tissues (Keller, 2002; Shindo, 2018). Across key stages, notochord formation in *Xenopu*s is mitosis independent (Rollins and Andrews, 1991), indicating that midline morphogenesis is largely driven by cell rearrangements.

### 3.2 Wnt/PCP control of CE

Non-canonical Wnt ligands and PCP components regulate CE by orienting cell behaviors and stabilizing mediolateral intercalation (Tada and Smith, 2000; Wallingford et al., 2000; Matsui et al., 2005). Across vertebrate models, PCP disruption typically yields shortened and widened body axes and defects in elongation of embryonic midline structures (Tada and Smith, 2000; Wallingford et al., 2000; Matsui et al., 2005; Voiculescu et al., 2007; Asai et al., 2024). These phenotypes support a direct mapping in which PCP-biased rearrangements both elongate the axis and generate macroscopic displacement fields. Within this regime, midline morphogenesis and large-scale cellular flows during gastrulation are tightly coupled. At the cellular level, this involves PCP-dependent polarization of actomyosin and adhesion dynamics, which orients neighbor exchange and stabilizes mediolateral intercalation (Shindo, 2018).

## 4 Amniotes: primitive streak formation and the unresolved role of Wnt/PCP in cellular flows

### 4.1 Primitive streak formation in an embryonic disc in avian embryo

Amniotes establish the earliest midline through the primitive streak (PS), a transient embryonic structure that serves as the site of cell ingression and germ-layer formation (Stern, 2004; Gilbert and Barresi, 2017). In the avian embryo, PS emergence and extension occur within an epiblast and are accompanied by prominent large-scale cellular flows in the anterior epiblast (named as ‘polonaise movements’) (Gräper, 1929; Wetzel, 1929). These flows constitute one of the characterized examples of tissue-scale collective cell movements in vertebrate gastrulation and provide a useful framework for testing how Wnt/PCP pathway is deployed in a clade-specific developmental context.

### 4.2 Wnt/PCP is essential for primitive streak morphogenesis

Multiple studies in chick embryos indicate that Wnt/PCP pathway is required for normal PS morphogenesis, with perturbations frequently producing a shorter and wider streak and defects in extension (Voiculescu et al., 2007; Asai et al., 2024). These phenotypes are consistent with impaired Wnt/PCP-mediated convergent extension, in which planar polarity within PS cells coordinates oriented cell behaviors and junctional remodeling to drive tissue narrowing and elongation through directional cell intercalation (Serrano Nájera and Weijer, 2020). Thus, in amniotes, the Wnt/PCP pathway is widely regarded as a key regulator of the morphology and extension of this initial midline structure.

### 4.3 What is (and is not yet) known about Wnt/PCP and cellular flows during PS development

Despite the clear requirement for the Wnt/PCP pathway in PS morphogenesis, the polonaise movements can still occur when the Wnt/PCP pathway is perturbed (Chuai et al., 2006; Asai et al., 2024). Flow generation and the bilateral topology of the flow field are not simply downstream readouts of PCP activity. On the other hand, these studies do not exclude important contributions of PCP to quantitative features of tissue-scale flow, such as speed distributions, spatial symmetry, temporal coordination, or the timing of transitions between flow modes, because these parameters are often not assessed as primary endpoints in PCP studies.

**FIGURE 1:**
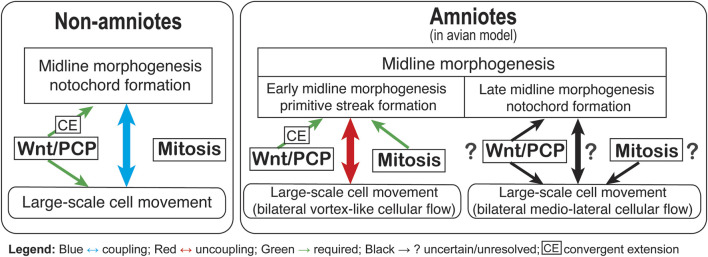
Summary model for stage- and clade-dependent relationships among Wnt/PCP, mitosis, tissue-scale cellular flows, and midline morphogenesis (avian amniote model). Left (Non-amniotes): During gastrulation in fish and amphibians, midline morphogenesis (notochord formation) is tightly linked to large-scale cell movement. Wnt/PCP is required (green arrows) for CE and for polarized collective rearrangements that underpin tissue elongation, consistent with a tightly coupled relationship between midline morphogenesis and tissue-scale movement (blue bidirectional arrow). Right (Amniotes; avian model): Midline morphogenesis is partitioned into an early phase (primitive streak formation) and a late phase (notochord formation). In the early phase, Wnt/PCP is required for primitive streak morphogenesis and CE, whereas large-scale vortex-like cellular flows (polonaise movements) can be partially uncoupled from streak morphogenesis (red bidirectional arrow). In the late phase, the extent to which Wnt/PCP and mitosis regulate notochord formation and associated bilateral medio-lateral cellular flows remains uncertain (black arrows with question marks).

Taken together, current studies support a modular relationship in which the Wnt/PCP pathway is essential for organizing the primitive streak formation but may not be the sole determinant of whether flows occur. Resolving this issue will require systematic, quantitative comparisons of flow fields under graded perturbations of PCP components, ideally coupled to markers of planar polarity and cell behaviors within the epiblast (Figure 1).

### 4.4 Decoupling experiments highlight open questions about Wnt/PCP function in amniote development

Perturbations that compromise cell division can strongly inhibit PS extension while still allowing the initiation of the polonaise movements (Cui et al., 2005; Saadaoui et al., 2020; Asai et al., 2025), indicating that early collective motion can arise even when robust streak morphogenesis is impaired. Conversely, ectopic axis induction can re-route flow fields and generate additional streak-like axes, demonstrating that flow patterns can be reprogrammed without abolishing streak induction (Asai et al., 2024). These findings support a modular view in amniotes and underscore an important open problem: Wnt/PCP is essential for streak morphogenesis, whereas the extent to which it shapes flow topology, robustness, and asymmetry during PS development remains to be determined.

### 4.5 After PS extension: a second window of midline morphogenesis with distinct mitosis sensitivity in amniotes

Whereas the Wnt/PCP pathway during non-amniote gastrulation is relatively well characterized, many open questions remain in amniotes, particularly in the post–PS extension window of midline morphogenesis, which exhibits distinct sensitivity to mitotic inhibition (Zhao et al., 2025). During PS extension in a chick embryo, mitosis contributes substantially to proper streak elongation, consistent with previous reports (Cui et al., 2005; Saadaoui et al., 2020; Asai et al., 2024). In contrast, after full PS extension, mitotic arrest can yield smaller embryos while preserving notochord extension and node regression, indicating that a substantial portion of axial midline morphogenesis can proceed with lower dependence on cell division (Figure 1) (Zhao et al., 2025). This window also highlights amniote embryo scaling and size-compensation behaviors (size reduction with cell hypertrophy), motivating functional tests of the Wnt/PCP requirement during early notochord morphogenesis using quantitative imaging readouts.

## 5 Conserved principles vs. species-specific implementations

A conserved principle across vertebrates is that Wnt/PCP provides a PCP module that biases cell behaviors (e.g., cell rearrangement, cell-shape changes, and cell division), in many contexts, supports tissue-scale morphogenesis and flow (Keller et al., 2000; Myers et al., 2002). The species-specific implementation of this module becomes especially clear during amniote gastrulation, where the earliest midline is built through the PS within the epiblast cell layer. In this amniote context, recent findings on pre-node LR asymmetry associated with polonaise movements in the early chick embryo indicate that tissue-scale cellular flows can provide an early physical readout of laterality upstream of a robust organizer-centric gene program (Asai et al., 2025). However, during amniote PS development, the relative contributions of PCP-regulated local remodeling and epiblast-wide tissue context to flow organization, stability, and asymmetry remain unresolved. Addressing this amniote-specific gap will require quantitative endpoints beyond PS length/width, including flow parameters and polarity readouts, together with stage-dependent analyses under graded PCP perturbations across PS initiation, extension, and the transition to post-PS stages.

Developmental architecture is a key determinant of how this conserved pathway should be interpreted across clades. In non-amniotes, Wnt/PCP-dependent CE often links PCP to axis elongation in a relatively direct manner (Wallingford et al., 2000; Matsui et al., 2005). In amniotes, by contrast, PS morphogenesis unfolds within an epithelial embryonic disc and coincides with prominent tissue-scale flows and stronger growth requirements, conditions that can partially decouple flow dynamics from specific morphogenetic outputs (Asai et al., 2024; Zhao et al., 2025). A central challenge in amniotes, therefore, is to identify which PCP outputs (e.g., junctional remodeling, CE directionality via directed intercalation, or biased cell division) dominate at particular stages of PS and post-PS midline development, and to determine how these outputs regulate key features of tissue-scale flows, including their organization, stability, and asymmetry.

## 6 Discussion

Focusing on amniote development, this section summarizes current insights into how the Wnt/PCP pathway interfaces with PS morphogenesis, tissue-scale flows, and early LR patterning during gastrulation. In amniotes, these processes unfold within an epithelial embryonic disc and can show partial uncoupling between specific morphogenetic outputs, motivating stage-resolved interpretations of pathway function.

A key open question in amniote gastrulation is what is a driving force of the polonaise movements, and how the Wnt/PCP pathway relates to these processes. Cellular flow initiation can occur even when PS extension is compromised, implying that vortical flows do not strictly require PS morphogenesis (Cui et al., 2005; Asai et al., 2024; Asai et al., 2025). This points to upstream determinants acting at the epiblast scale, while leaving open the possibility that Wnt/PCP primarily refines or patterns these movements rather than serving as the sole trigger. A key next step is to define minimal requirements for sustained vortices using quantitative flow analysis combined with graded perturbations of PCP components, with staging that separates effects on PS geometry from effects on the flow field.

From an evolutionary perspective, these observations also motivate a layered model for early midline morphogenesis in amniotes. The PS represents an evolutionarily derived midline structure that was added to the vertebrate developmental program, and its extension may therefore incorporate a proliferation-dependent module in addition to the conserved Wnt/PCP-mediated CE machinery (Wei and Mikawa, 2000; Zhao et al., 2025). This framing helps reconcile stage-specific sensitivities: PS extension can be strongly affected by mitotic inhibition even when large-scale flows can still be initiated, consistent with partial uncoupling between streak morphogenesis and flow onset.

A second open question is when and how LR bias first appears within the polonaise movements. Quantitative analyses indicate that right-side dominance emerges reproducibly around ∼6 h after motion onset and can persist under mitotic arrest (Asai et al., 2025), placing detectable LR bias upstream of a mature organizer-centric gene program. Multiple mechanisms could underlie this bias, including intrinsic cellular chirality, subtle early molecular asymmetries, or an asymmetric tissue context. Discriminating among these possibilities will require sensitive mapping of LR differences in flow parameters and targeted perturbations to test whether the Wnt/PCP pathway contributes to bias formation, bias amplification, or the robustness of bias expression.

Placing these amniote-specific questions in a broader comparative context helps distinguish conserved principles from species-specific implementations of the Wnt/PCP pathway. In non-amniotes, Wnt/PCP-dependent CE and axial/midline elongation are often more tightly coupled, providing a relatively direct link between planar polarity at the cellular level and tissue-scale axis extension. In amniotes, by contrast, primitive streak morphogenesis occurs within a disc-shaped epiblast alongside prominent tissue-scale flows and stronger growth requirements, conditions that can distribute control across multiple modules and allow partial uncoupling between flow dynamics and specific morphogenetic outputs. This comparison motivates quantitative endpoints in amniotes that go beyond PS length/width to include flow metrics and polarity readouts, enabling more direct cross-clade tests of which aspects of morphogenesis are Wnt/PCP-dependent.

Together, these issues motivate a stage-dependent view of Wnt/PCP function across amniote midline development. PCP perturbation can alter PS geometry without abolishing large-scale flows, suggesting that pathway activity is not equivalent to flow presence. Informative tests should quantify how PCP perturbations affect the symmetry, timing, and reproducibility of flows while monitoring planar polarity and junctional/cytoskeletal readouts in the epiblast. Whether post-PS notochord elongation and node regression are PCP dependent remains unresolved (Zhao et al., 2025). Resolving these links will unify models among vertebrates.

## 7 Conclusion

Across vertebrates, the Wnt/PCP pathway is a conserved organizer of planar polarization, however, its developmental roles diverge with embryonic architecture and stage (Figure 1). In non-amniotes, Wnt/PCP-regulated CE provides a relatively direct, largely proliferation-independent route to midline elongation across key gastrulation windows. By contrast, in amniotes, particularly in avians, PS-based gastrulation introduces an early proliferation-sensitive phase in which the Wnt/PCP pathway contributes to streak geometry, while the polonaise movements can still initiate and persist even when PS extension is impaired. In the chick embryo, these flows can also exhibit pre-node LR asymmetry, indicating a detectable laterality bias upstream of a mature organizer-centric gene program. After PS extension, a second phase follows in which axial morphogenesis becomes comparatively mitosis resistant and embryos can scale down while preserving core morphogenetic trajectories. Taken together, these comparisons support a stage-aware framework in which a conserved signaling modules is deployed within clade-specific developmental architectures, producing distinct coupling relationships among signaling, proliferation-dependent tissue growth, tissue-scale flows, and early laterality.
